# Comprehensive Analysis to Identify the Epithelial–Mesenchymal Transition-Related Immune Signatures as a Prognostic and Therapeutic Biomarkers in Hepatocellular Carcinoma

**DOI:** 10.3389/fsurg.2021.742443

**Published:** 2021-10-15

**Authors:** Guozhi Wu, Yuan Yang, Yu Zhu, Yemao Li, Zipeng Zhai, Lina An, Min Liu, Ya Zheng, Yuping Wang, Yongning Zhou, Qinghong Guo

**Affiliations:** ^1^The First Clinical Medical College, Lanzhou University, Lanzhou, China; ^2^Department of Gastroenterology, The First Hospital of Lanzhou University, Lanzhou, China; ^3^Gansu Key Laboratory of Gastroenterology, Lanzhou University, Lanzhou, China; ^4^Department of Hematology, the First Hospital of Lanzhou University, Lanzhou, China

**Keywords:** hepatocellular carcinoma (HCC), epithelial-mesenchymal-transition (EMT), overall survival (OS), immune infiltration, chemosensitivity

## Abstract

**Background:** Hepatocellular carcinoma (HCC) is a highly heterogeneous disease with the high rates of the morbidity and mortality due to the lack of the effective prognostic model for prediction.

**Aim:** To construct a risk model composed of the epithelial–mesenchymal transition (EMT)-related immune genes for the assessment of the prognosis, immune infiltration status, and chemosensitivity.

**Methods:** We obtained the transcriptome and clinical data of the HCC samples from The Cancer Genome Atlas (TCGA) and The International Cancer Genome Consortium (ICGC) databases. The Pearson correlation analysis was applied to identify the differentially expressed EMT-related immune genes (DE-EMTri-genes). Subsequently, the univariate Cox regression was introduced to screen out the prognostic gene sets and a risk model was constructed based on the least absolute shrinkage and selection operator-penalized Cox regression. Additionally, the receiver operating characteristic (ROC) curves were plotted to compare the prognostic value of the newly established model compared with the previous model. Furthermore, the correlation between the risk model and survival probability, immune characteristic, and efficacy of the chemotherapeutics were analyzed by the bioinformatics methods.

**Results:** Six DE-EMTri-genes were ultimately selected to construct the prognostic model. The area under the curve (AUC) values for 1-, 2-, and 3- year were 0.773, 0.721, and 0.673, respectively. Stratified survival analysis suggested that the prognosis of the low-score group was superior to the high-score group. Moreover, the univariate and multivariate analysis indicated that risk score [hazard ratio (HR) 5.071, 95% CI 3.050, 8.432; HR 4.396, 95% CI 2.624, 7.366; *p* < 0.001] and stage (HR 2.500, 95% CI 1.721, 3.632; HR 2.111, 95% CI 1.443, 3.089; *p* < 0.001) served as an independent predictive factors in HCC. In addition, the macrophages, natural killer (NK) cells, and regulatory T (Treg) cells were significantly enriched in the high-risk group. Finally, the patients with the high-risk score might be more sensitive to cisplatin, doxorubicin, etoposide, gemcitabine, and mitomycin C.

**Conclusion:** We established a reliable EMTri-genes-based prognostic signature, which may hold promise for the clinical prediction.

## Introduction

Hepatocellular carcinoma (HCC) has become the sixth most common carcinomas with a high mortality rate worldwide (1, 2). Asians and Pacific Islanders have the highest morbidity (4.7 per 100,000 people) and mortality (2.8 per 100,000 people) rates among the young adults, whereas Blacks have the highest morbidity (26.9 per 100,000 people) and mortality (18.6 per 100,000 people) rates among the middle-aged persons during the years 2000–2010 (3). The chronic inflammation, induced by infection of hepatitis C virus (HCV) or hepatitis B virus (HBV), alcohol, non-alcoholic fatty liver disease (NAFLD), and so on, is the main mechanism driving the occurrence and development of liver cancer (4–8). However, many patients with HCC with high-risk factors are often diagnosed with advanced status, resulting in a 5-year survival of only about 10% (9).

Epithelial–mesenchymal transition (EMT) is a process in which the epithelial cells lose the connection and polarity during the embryogenesis, leading to a migrating mesenchymal phenotype (10). It is widely accepted that EMT plays crucial roles in the different biological and pathological processes of HCC including tumor progression, metastasis after chemotherapy treatments, and anticancer drugs resistance (11–13). Among them, EMT tumor drug resistance gradually arouses the attention of the researchers. Increasing studies have reported that EMT is involved in the drugs resistance in the plenty of cancers such as HCC (14), breast cancer (15, 16), pancreatic cancer (17), and bladder cancer (18). With respect to the mechanism of EMT-mediated chemotherapy resistance, several studies have found that transforming growth factor-β (TGF-β) (19, 20), Wnt (21, 22), Hedgehog (21, 23), p53 (24), and phosphatidylinositol 3-kinase (PI3K)/Akt (22, 25) signaling pathways involved in drugs resistance are closely related to EMT. In addition, the development of cell resistance to drug-induced apoptosis and the local effect of tumor microenvironment (TME) are also two factors driving EMT-mediated drug resistance (26). Through modulating the course of EMT and the immune response, the TME-related exosomes may also mediate the drug resistance process (27).

Some genes have been found to be involved in EMT. For example, the activation of TGF-β downstream signals is a key molecular event in the induction of EMT (19, 20, 28). Besides, the stimulation of fibroblast-derived CXCL14/ACKR2 pathway proceeds the EMT process, as demonstrated by a reduction of the expression level of the epithelial marker E-cadherin and increased the expression of the mesenchymal biomarkers (N-cadherin and vimentin) (29, 30). Mounting data have already found that the EMT-related genes are associated with the onset and progression of the tumors. In lung cancer, midkine (MDK) enhances the EMT capability of the cancer cells by TGF-β, Wnt, and Notch2 signaling pathways (31–33). An experiment has demonstrated that the suppression of ficolins (FCNs) can upregulate TGF-β, which, in turn, activates the downstream pathways to inhibit EMT in HCC (34). Additionally, it is found in colorectal cancer that secreted phosphoprotein 1 (SPP1) can promote the metastasis of the cancer cells by activating EMT (35).

Various novel signatures have been established recently to uncover the potential mechanisms of cancer and the application prospects of the biomarkers associated with the onset of tumor progression (36–38). In this study, we established a prognostic model based on differentially expressed EMT-related immune genes (EMTri-genes) to perform the prognosis prediction in the patients with HCC. Next, the functional enrichment analyses were utilized to explore the underlying regulatory mechanisms of the signature. Moreover, the correlations between the signature and immune infiltration status and chemosensitivity were assessed by the single-sample gene set enrichment analysis (ssGSEA) and the R package pRRophetic, respectively.

## Methods

### Retrieval and Download of the Data From the TCGA-LIHC and the ICGC Liver Cancer - RIKEN, Japan (LIRI-JP) Datasets

The RNA sequencing and clinical data for the patients with HCC were retrieved and downloaded from The Cancer Genome Atlas-Liver Hepatocellular Carcinoma (TCGA-LIHC) dataset (https://tcga-data.nci.nih.gov/tcga/) and the International Cancer Genome Consortium (ICGC) portal (https://dcc.icgc.org/projects/LIRI-JP), respectively. The former dataset was regarded as a training cohort with 365 cases, while another considered as a validation cohort of 231 patients. Next, the EMT-related genes were obtained from the Molecular Signatures Database (MSigDB) (http://software.broadinstitute.org/gsea/msigdb). Meanwhile, immune-related genes list was downloaded from the ImmPort website (http://www.immport.org). Thereafter, 576 of EMT-related genes and 1,626 of immune-related genes were identified in this study (Appendices 1, 2).

### Screening out EMT-Related Immune Genes

To screen for the EMTri-genes, the Pearson correlation coefficient was set to be larger than 0.4 and *p* < 0.001. Subsequently, R package limma was utilized to perform the identification of the differentially expressed EMTri-genes (DE-EMTri-genes) by setting up a threshold of log2 fold change (FC) ≥ 2 along with a false discovery rate (FDR) < 0.05 in the training cohort.

### Establishment of a Risk Model for the Direct Evaluation of the Risk Scores

First, the univariate Cox analysis was used to screen for the overall survival (OS)-related genes. In addition, the protein–protein interaction (PPI) and gene–gene interaction (GGI) network based on these OS-related genes were constructed by the Search Tool for the Retrieval of Interacting Genes/Proteins (STRING) database (http://string-db.org/) in order to explore the potential regulatory relationships of these genes. Subsequently, we performed the Lasso regression, with 10-fold cross-validation and *p*-value of 0.05, for variable selection based on the significant results of the univariate analysis. The Lasso regression run for a total of 1,000 cycles and the random stimulus for each cycle was set to 1,000. Genes with frequency more than 100 were chosen to perform the Cox proportional hazards regression so as to construct the prognostic model. We calculated the area under the curve (AUC) value of each model and connected each AUC value into a curve. The calculation would not be suspended until the curve reached its maximum value, at which point the model would be listed as optimal. Additionally, we plotted the 1-, 2-, and 3-year receiver operating characteristic (ROC) curves to reflect the prognostic accuracy. Next, the risk scores [formula: $\overset{n}{\underset{i=1}{\sum }}$ (expression level ofgene*regression coefficient)] were calculated for each patient to distinguish between the high- and low-risk groups in accordance with the median of the risk scores. Besides, the principal component analysis (PCA) and t-statistic stochastic neighborhood embedding (t-SNE) were utilized for the visualization of the samples distribution in the two groups by using R package Stats and Rtsne, respectively. The Kaplan–Meier method was conducted to verify the survival duration differences between the two groups. Accordingly, the survival curves and forest plot were drawn. A logistic regression model was established by using the rms package in R software and the nomogram was used for visualization. The variables included in the model construction were age, gender, grade, stage, T, N, and M, respectively. Subsequently, the calibration curve was drawn to make a comparison of the predicted and real results.

### Stratified Analysis

The stratified survival analyses were conducted based on the several clinicopathological characteristics including the age; sex; tumor, node, and metastasis (TNM) stages; and clinical stages. The HCC samples were classified into T1/T2 (the diameter of isolated tumor >2 cm, with vascular invasion; or multiple tumors, the diameter <5 cm) and T3/T4 (single or multiple tumors, involving the main branches of the portal vein or hepatic vein) according to the TNM system of the American Joint Committee on Cancer (AJCC), eighth edition. Moreover, the patients with HCC were categorized as N0 (node negative) and N1 (regional lymph node metastasis) according to the lymph node metastasis. Based on the distant metastasis, the patients with HCC were divided into M0 (no distant metastasis) and M1 (with distant metastasis). In addition, the patients with HCC were also categorized as stage I/II (early stage) and stage III/IV (advanced stage).

### Enrichment Analysis

Differentially expressed genes (DEGs) were entered into the Gene Ontology (GO) (http://www.geneontology.org/) and the Kyoto Encyclopedia of Genes and Genomes (KEGG) (http://www.genome.jp/kegg/) websites to obtain the enriched GO terms and significant KEGG pathways. These analyses were performed by the clusterProfiler package implemented in the R package (log2FC > 1.5 and *p* < 0.05 were the critical values).

### Tumor Immune Infiltration Status Assessment

The ssGSEA based on the R package gene set variation analysis (GSVA) was used to investigate the enrichment levels of the immune cells and corresponding immune-related functions between the two risk groups. The HCC samples used for analysis were extracted from the TCGA-LIHC and the LIRI-JP dataset, respectively.

### Evaluation of Constructed Model in Clinical Practice

The half-maximal inhibitory concentration (IC50) was calculated by using the R package pRRophetic for administrating the chemotherapeutic drugs, such as cisplatin, doxorubicin, gemcitabine, mitomycin C, vinblastine, and sorafenib, to predict the chemosensitivity in the different risk score groups. Subsequently, the Wilcoxon signed-rank test was adopted to compare the IC50 between the two groups.

## Results

### Screening for EMT-Related Immune Genes and Construction of the Risk Models for the Evaluation of Prognosis

**Figure 1:**
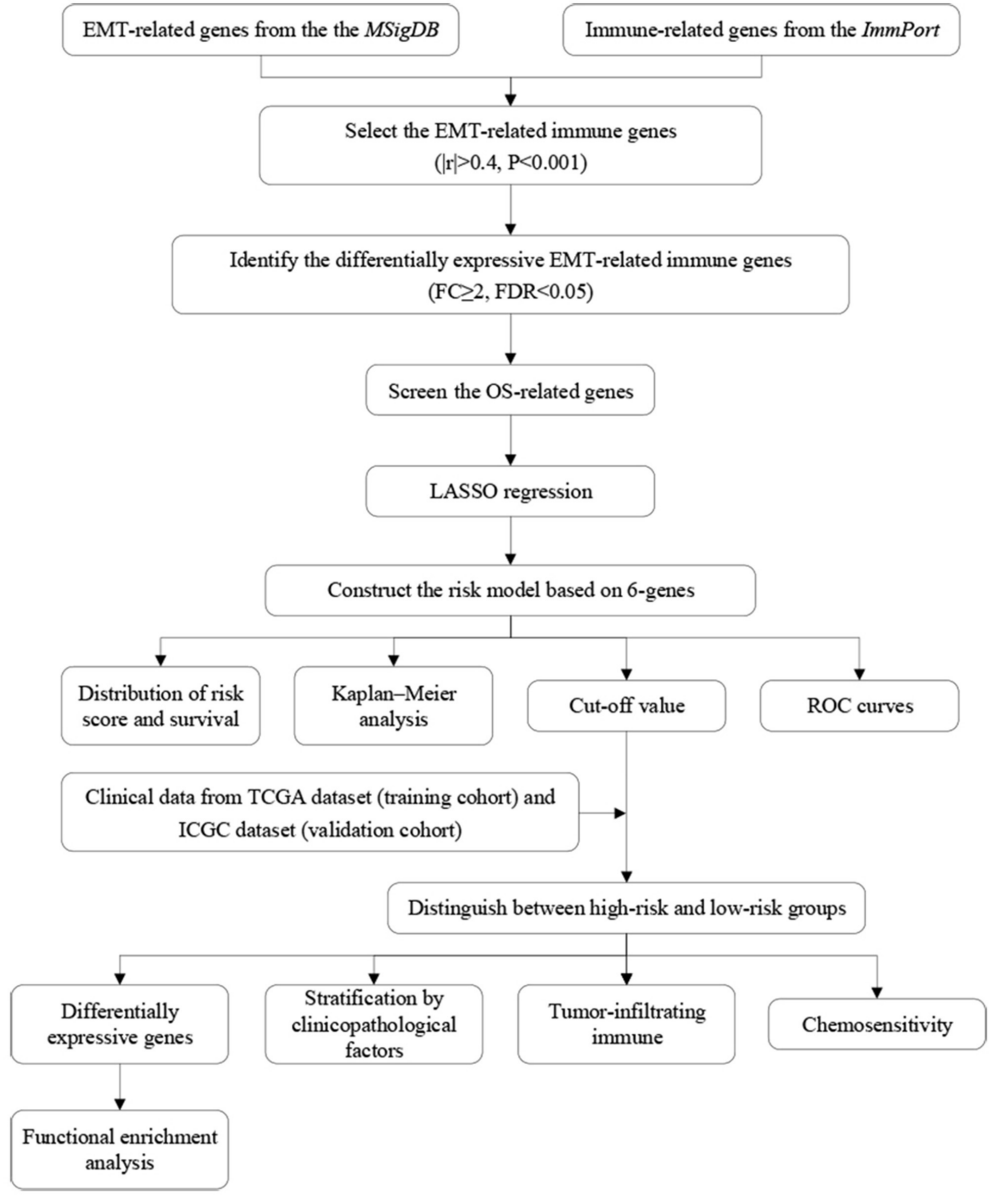
Flow diagram of the study.

**Figure 2:**
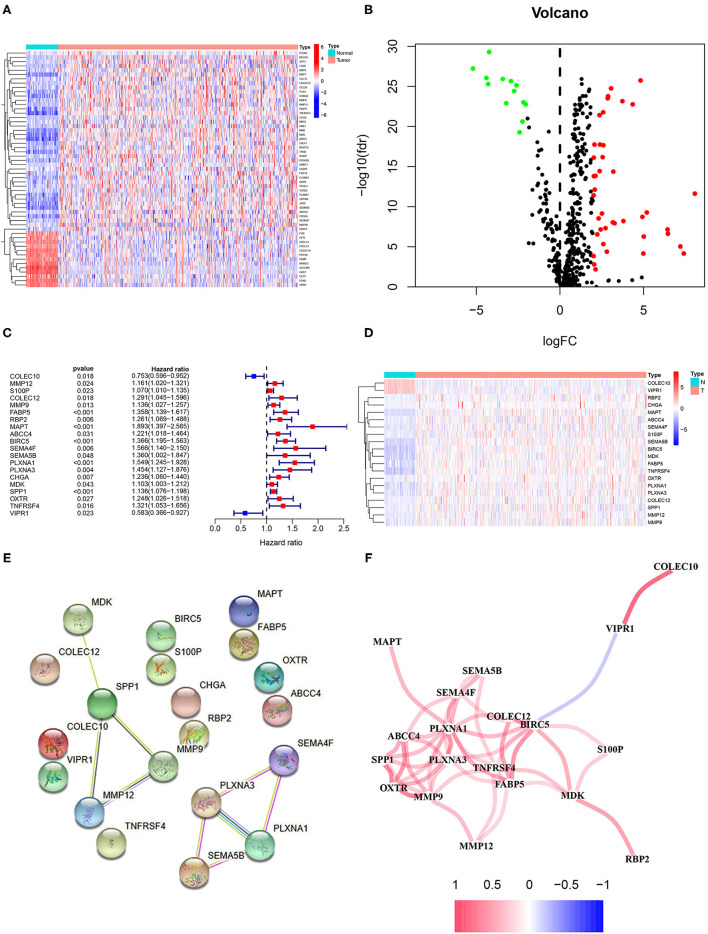
Identification of the epithelial–mesenchymal transition (EMT)-related immune genes with the differential expression and overall survival (OS)-related genes as well as plotting of the protein–protein interaction (PPI) and gene–gene interaction (GGI). **(A)** The heatmap and **(B)** volcano plots of the differentially expressed (DE)-EMT-related immune genes in hepatocellular carcinoma (HCC). **(C)** The forest and **(D)** heatmap plots of the OS-related genes based on the univariate Cox regression analysis. Network plot of **(E)** the PPI and **(F)** GGI.

**Figure 3:**
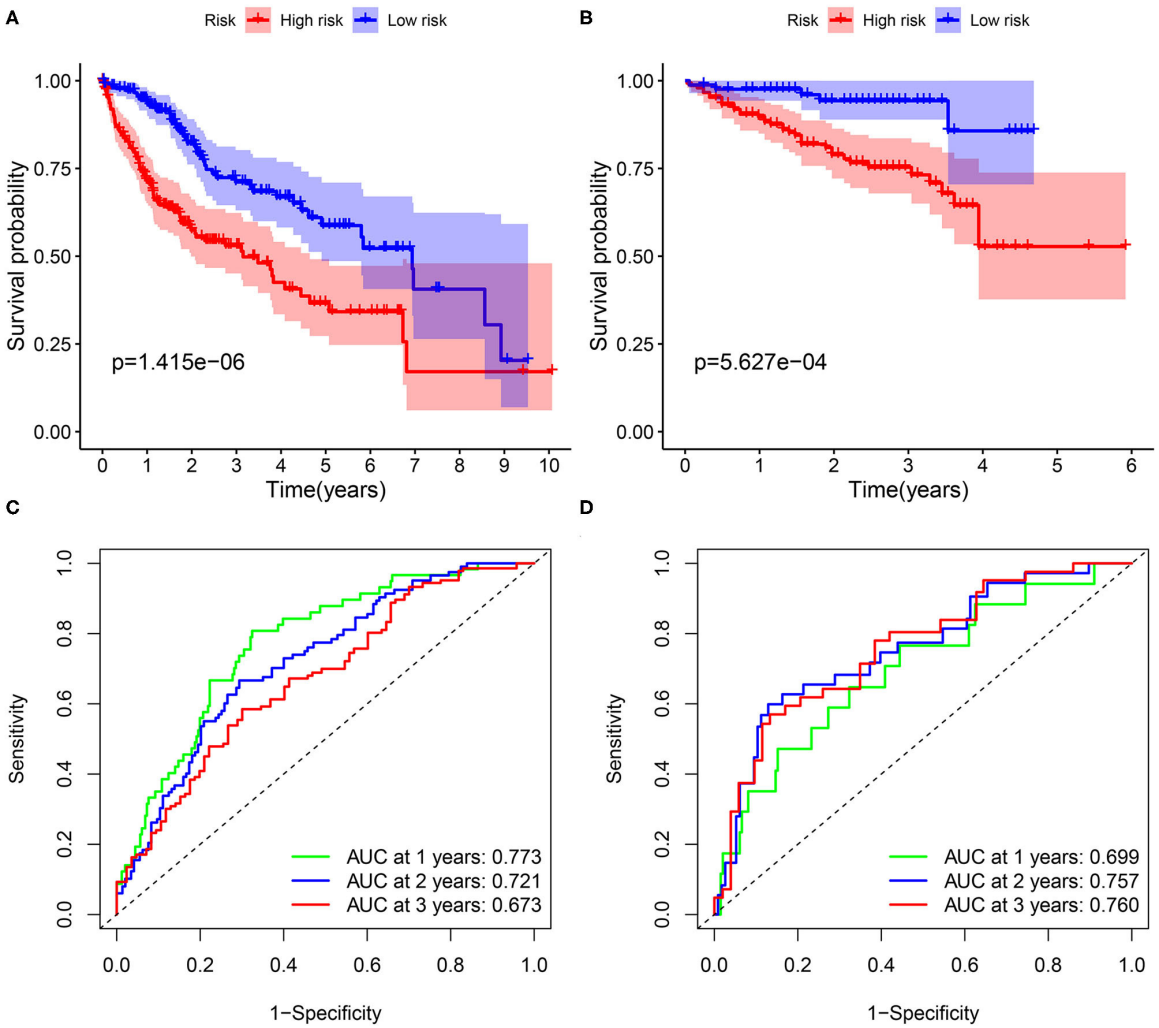
The Kaplan–Meier analysis and the construction of prognostic model based on *FABP5, MAPT, BIRC5, PLXNA1*, and *SPP1* genes. The survival curves in the **(A)** training cohort and **(B)** validation cohort. The 1-, 2-, and 3-year receiver operating characteristic (ROC) curves for assessing the prognostic performance of the gene signature in **(C)** The Cancer Genome Atlas (TCGA) dataset and **(D)** the International Cancer Genome Consortium (ICGC) dataset.

**Figure 4:**
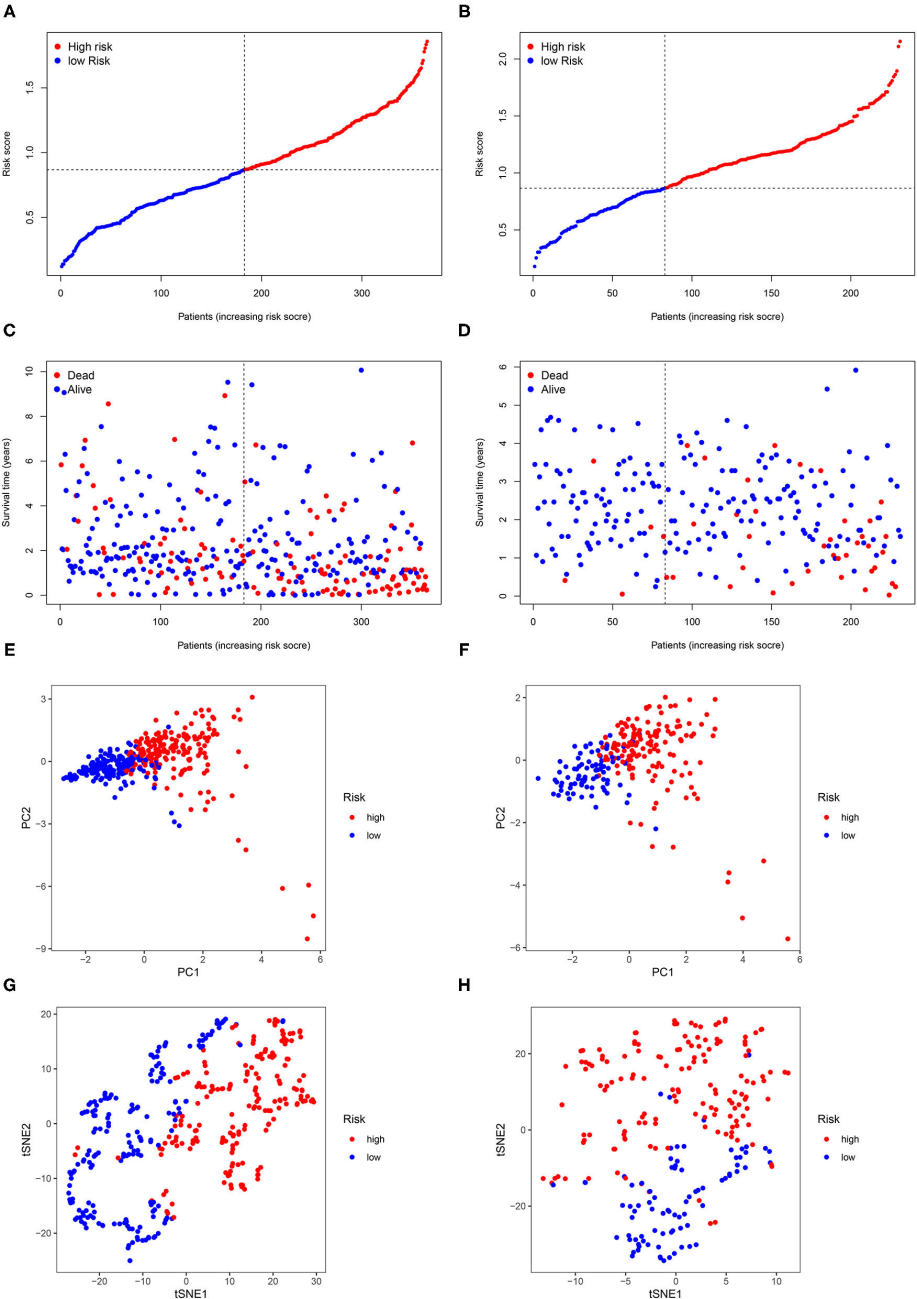
The risk score and survival time distribution of each patient. Risk score distribution in **(A)** the TCGA dataset and **(B)** the ICGC dataset. Survival time distribution in **(C)** the TCGA dataset and **(D)** principal component analysis (PCA) in **(E)** the TCGA dataset and **(F)** the ICGC dataset. t-statistic stochastic neighborhood embedding (t-SNE) in **(G)** the TCGA dataset and **(H)** the ICGC dataset.

**Figure 5:**
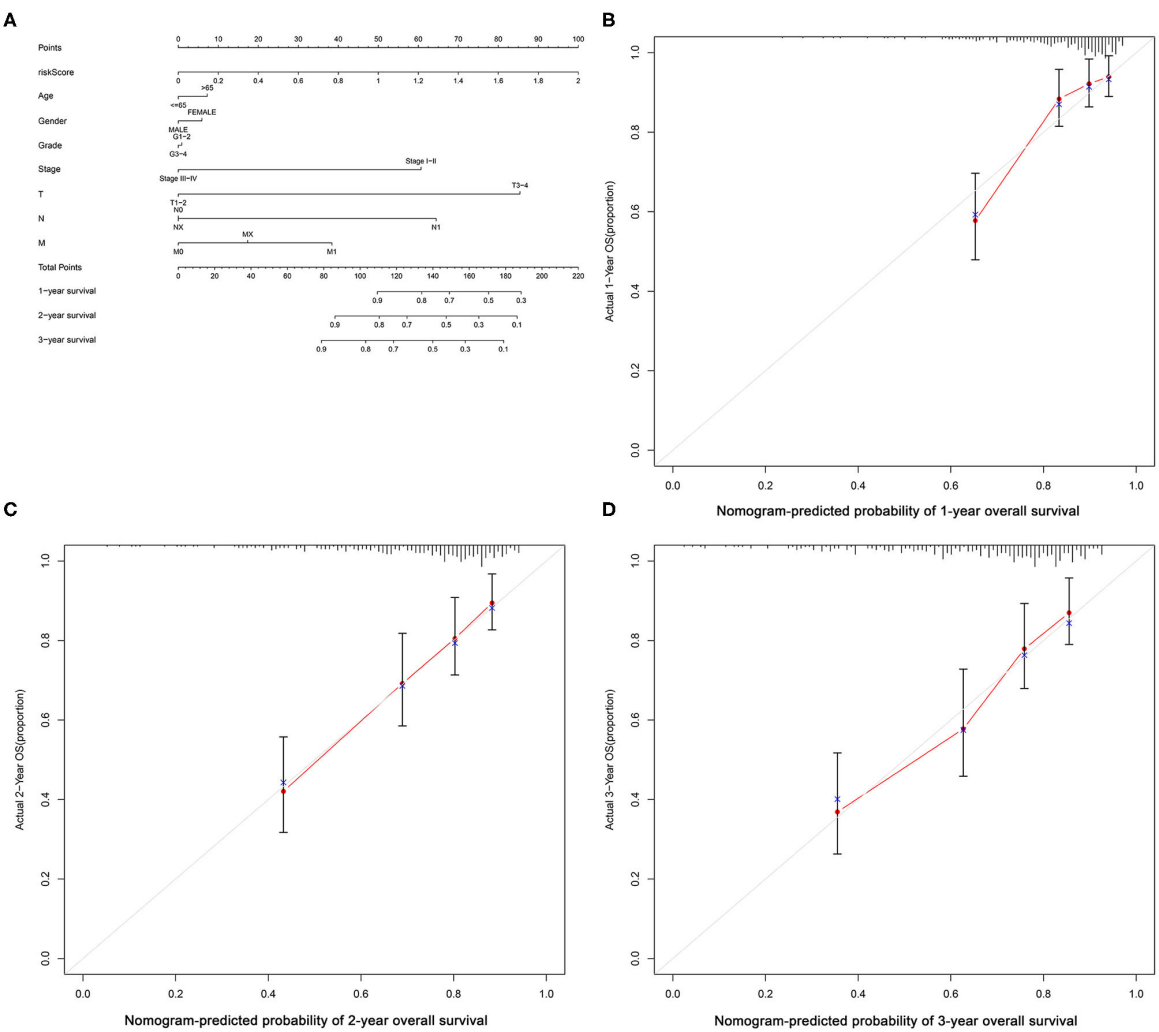
Nomogram of the logistic regression model and calibration curves. **(A)** Nomogram; nomogram-predicted probability of **(B)** 1-year, **(C)** 2-year and **(D)** 3-year OS.

The flow diagram of this study is presented in Figure 1. The Pearson correlation coefficient analysis was utilized to identify the EMTri-genes. As a result, 456 of EMTri-genes were obtained from the TCGA-LIHC cohort and then 55 of the DE-EMTri-genes were identified (Figures 2A,B). Next, the OS-related genes with significant difference were identified via the univariate Cox regression analysis and included in the Lasso regression analysis to establish the prognostic models based on the *RBP2, MAPT, BIRC5, PLXNA1, CHGA*, and *SPP1* genes (Figures 2C,D). The risk score formula is as follows: risk score = 0.013023617745279 × messenger RNA (mRNA) expression level of *RBP2*+ 0.262079957422771 × mRNA expression level of *MAPT*+ 0.141033589913412 × mRNA expression level of *BIRC5*+ 0.0443117549486775 × mRNA expression level of *PLXNA1*+ 0.0236121577329352 × mRNA expression level of *CHGA*+ 0.0594398057536237 × mRNA expression level of *SPP1*. Network of the PPI and GGI was shown in Figures 2E,F. The risk scores for the patients with HCC in the training cohort were calculated and the patients were then separated into the different risk groups according to the median score. Subsequently, the Kaplan–Meier survival analysis suggested that, compared to the high-risk group, the low-risk group associated with longer OS (Figure 3A). The ROC curves for 1-, 2-, and 3- year were drawn and the corresponding AUC values were 0.773, 0.721, and 0.673, respectively (Figure 3C). The risk score and survival time distribution of each patient were illustrated in Figures 4A,C. For the enhanced visualization property, the PCA and t-SNE methods were adopted to provide the good separation display effects (Figures 4E,G). The logistic regression model constructed by the age, gender, grade, stage, T, N, and M was visualized by the nomogram (Figure 5A). The calibration curves showed sufficient consistency between the predicted and real findings (Figures 5B–D).

### Verifying Prognostic DE-EMT-Related Immune Gene Signature Based on the International Cancer Genome Consortium Dataset

The ICGC dataset that serves as a validation cohort was utilized to confirm the prognostic value, demonstrated by the TCGA database, of DE-EMTri-genes-based signature. Meanwhile, the risk scores were also calculated for all the patients in the validation dataset to distinguish between the different risk groups. The survival curves showed an increased survival time in low-risk group, which was in line with the training cohort (Figure 3B). Lastly, it was concluded that 1-, 2-, and 3-year AUC of the EMTri-genes graded as 0.699, 0.757, and 0.760 in the validation results (Figure 3D), indicating a powerful predictive capacity of the signature. Similarly, the risk score and survival status distribution of the patients with HCC were plotted as well as the PCA and t-SNE methods were performed and the results were consistent with that of the TCGA dataset (Figures 4B,D,F,H).

### Clinicopathological Characteristics Analysis

**Figure 6:**
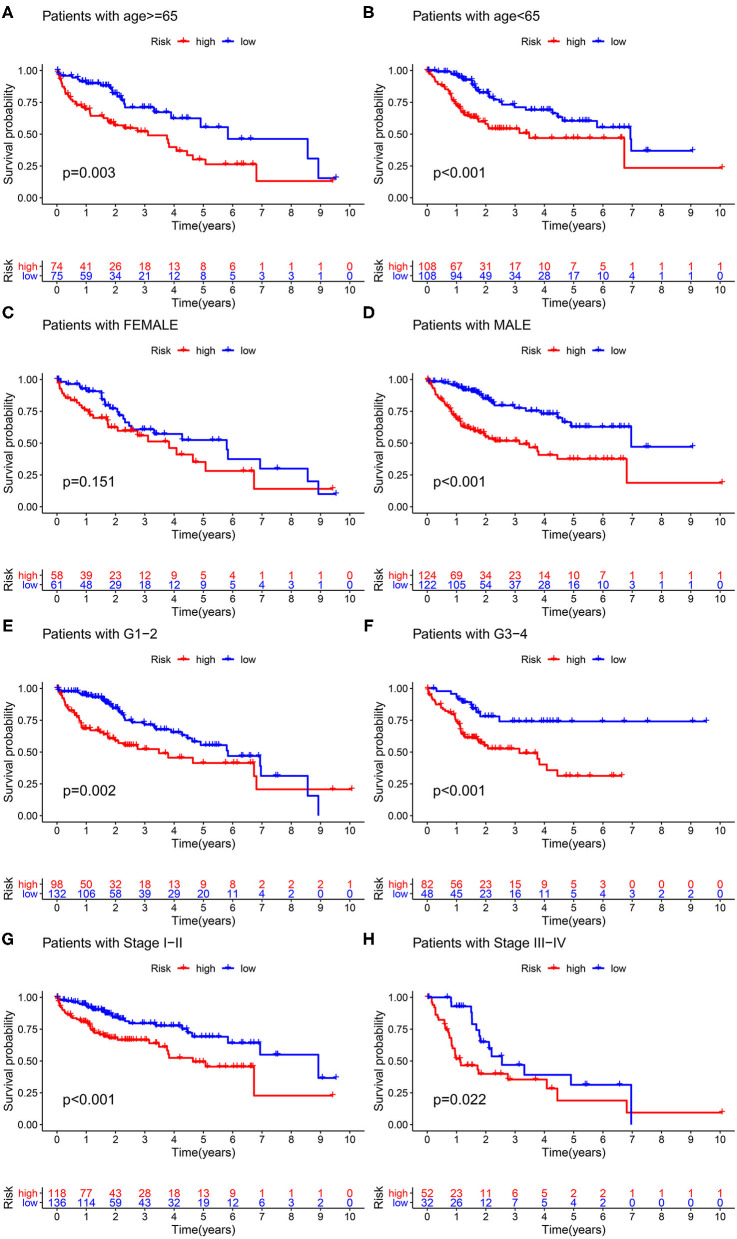
Clinicopathological characteristics analysis. Survival probability stratified by age **(A,B)**, gender **(C,D)**, grade **(E,F)**, and stage **(G,H)**.

As illustrated in Figure 6, significant survival differences between the two groups, stratified by the various clinicopathological characteristics, including age ≥ 65 years (*p* < 0.003), age < 65 years (*p* < 0.001), male (*p* < 0.001), G1–G2 (*p* = 0.002), stage I–II (*p* < 0.001), and stage III–IV (*p* = 0.022), were observed. The corresponding validation results were displayed in Supplementary Figure S3.

### Assessment of the Independent Prognostic Value of These Six Genes Status for Overall Survival

**Figure 7:**
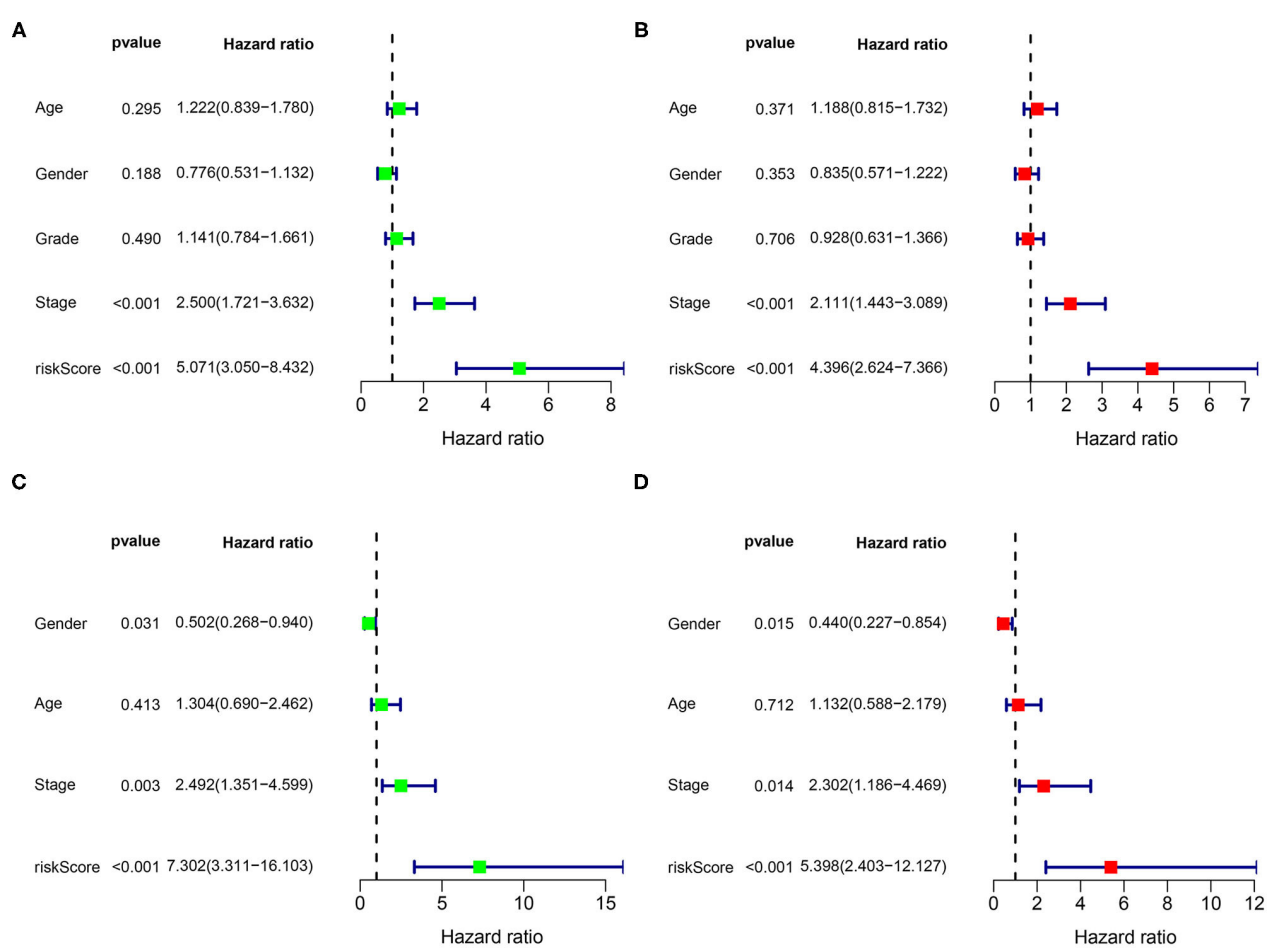
Univariate and multivariate analysis for the identification of the predictive factors. The forest plots of **(A)** the univariate and **(B)** multivariate results show that the risk score [hazard ratio (HR) 5.071, 95% CI 3.050, 8.432; HR 4.396, 95% CI 2.624, 7.366; *p* < 0.001] and stage (HR 2.500, 95% CI 1.721, 3.632; HR 2.111, 95% CI 1.443, 3.089; *p* < 0.001) are associated with poor prognosis in the TCGA dataset. The forest plots of **(C)** the univariate and **(D)** multivariate analysis in the ICGC dataset.

Risk score was identified as an independent predictive parameter between the different risk groups according to the univariate and multivariate Cox regression analysis. The univariate analysis illustrated a significant correlation between the risk score and OS in the training and validation cohorts (HR 5.071, 95% CI 3.050, 8.432, *p* < 0.001; HR 7.302, 95% CI 3.311, 16.103, *p* < 0.001; Figures 7A,C). In the multivariate analysis that excluded the confounding factors, a significant association between the risk score and OS was still observed (HR 4.396, 95% CI 2.624, 7.366, *p* < 0.001; HR 5.398, 95% CI 2.403, 12.127, *p* < 0.001; Figures 7B,D).

### Enrichment Analysis to Identify the DE-EMT-Related Immune Function Signatures

**Figure 8:**
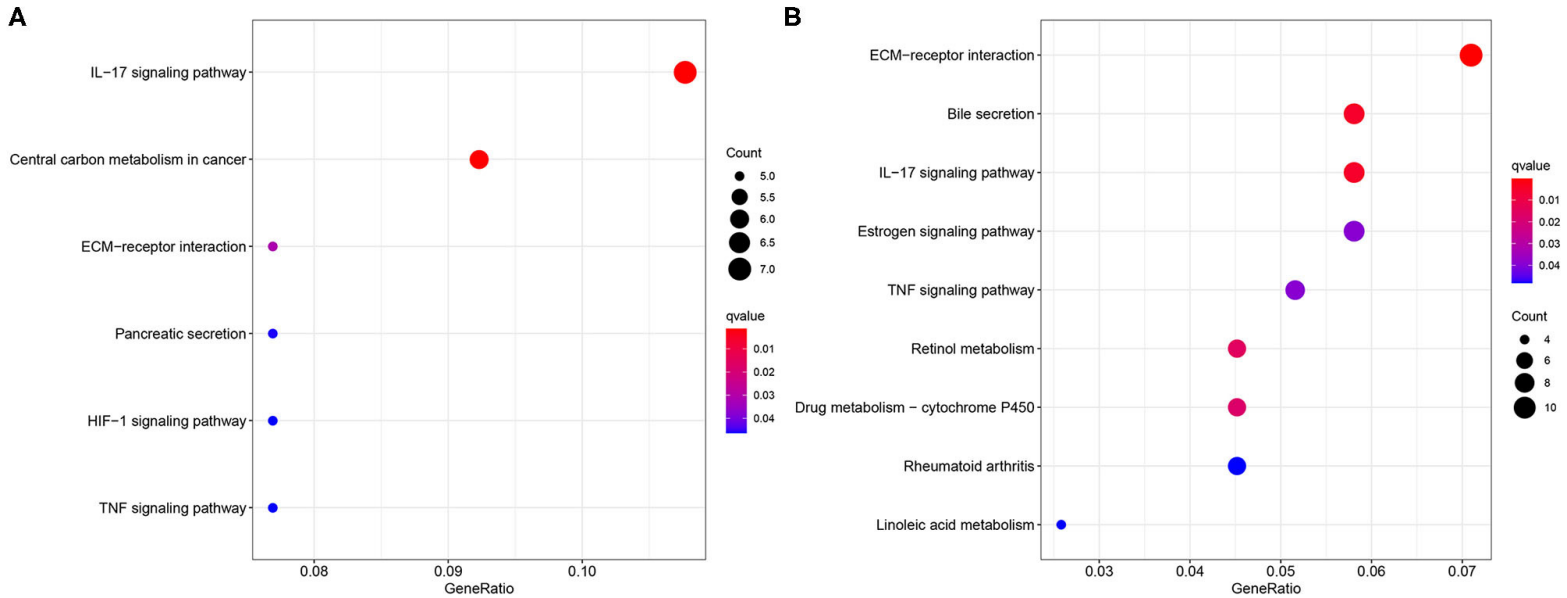
Enrichment of the Kyoto Encyclopedia of Genes and Genomes (KEGG) analysis. Bubble plot of the KEGG analysis based on **(A)** the TCGA dataset and **(B)** the ICGC dataset.

The GO and KEGG enrichment analyses were conducted to explore the biological process and signaling pathway of the DEGs between the different risk groups. As a result, the GO analysis revealed that the DEGs significantly enriched in extracellular matrix (ECM) structural constituent and calcium-dependent protein binding according to the TCGA (Supplementary Figure S1) and the ICGC datasets (Supplementary Figure S2). In addition, the ECM–receptor interaction and interleukin-17 (IL-17) signaling pathway were found to be with marked significance through the KEGG pathway enrichment analysis based on the two cohorts (Figures 8A,B).

### Immune Infiltration Status Analysis

**Figure 9:**
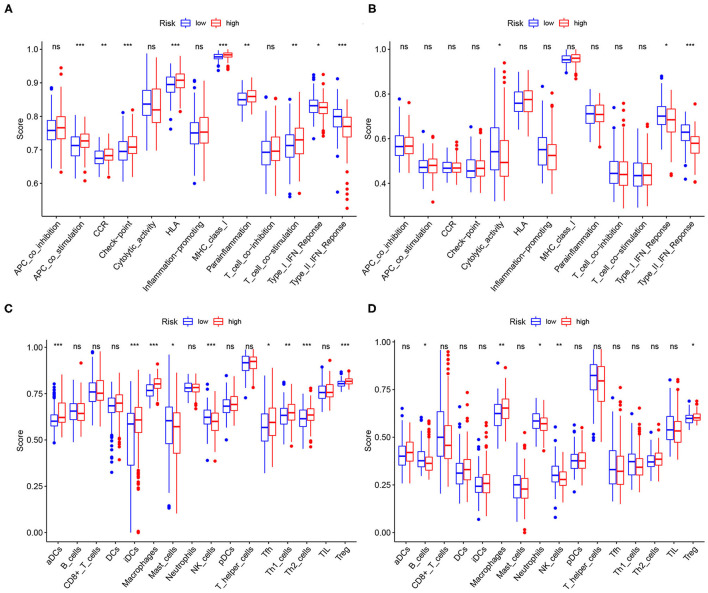
Relationships between the risk score, immune infiltration cells, and immune functions. The high-risk scores were positively correlated with infiltration of the macrophages, natural killer (NK) cells, and regulatory T (Treg) cells both in **(A)** the TCGA dataset and **(B)** the ICGC dataset. The high-risk scores were positively correlated with type I IFN response and type II IFN response both in **(C)** the TCGA dataset and **(D)** the ICGC dataset.

To assess the relationship between the signature and TME, the infiltrating levels of the immune cells and specified immune-related functions were analyzed via the ssGSEA based on the R package GSVA. Combined with the training and validation cohorts, the macrophages, natural killer (NK) cells, and regulatory T (Treg) cells occurred significantly more often in the tissues of the high-risk group, suggesting that these immune cells might be involved in the onset of the cancer progression (Figure 9A). Meanwhile, the results of immune function enrichment analysis demonstrated that the type I interferon (IFN-I) response and type II IFN (IFN-II) response were correlated with the high risk scores (Figure 9C), indicating that the immune cells probably exert their roles through these two pathways. The validation results were presented in Figures 9B,D.

### Evaluation of Chemosensitivity Based on the Constructed Risk Assessment Model

To evaluate the chemosensitivity differences between the two groups, the R package pRRophetic was utilized to calculate the IC50 value. In the high-risk group, cisplatin, doxorubicin, gemcitabine, and mitomycin C have lower IC50 values (i.e., higher chemosensitivity) (Supplementary Figures S4A–D). However, the IC50 of vinblastine was higher, suggesting that the high-risk population was less sensitive to chemotherapy with this drug (Supplementary Figure S4E). Additionally, no significant difference of chemosensitivity for sorafenib between the two risk groups was observed (Supplementary Figure S4F).

## Discussion

Hepatocellular carcinoma refers to the malignancies in the liver and the multiple factors play vital roles in the process of pathogenesis. The new diagnosed cases and mortality of HCC are gradually increasing worldwide (39). Moreover, due to the late detection, the survival time of the patients with HCC is significantly shortened. Therefore, there is an urgent need to find out the biomarkers related to the clinicopathological signatures and prognosis of HCC to help in the early diagnosis of HCC. In this study, we constructed a risk model based on the signatures of the EMTri-genes and provided a favorable performance to evaluate the corresponding prognostic value, immune infiltration status, and chemosensitivity to HCC.

Previous studies have established the prognostic models grounded on the different immune-related genes (40, 41), but the signatures are not convenient for the clinical application because of too many genes involved in the establishment of the model. In this study, *RBP2, MAPT, BIRC5, PLXNA1, CHGA*, and *SPP1* were identified as the OS-related genes and used for modeling. An *in vitro* test revealed that the high expression of *RBP2*was correlated with the poor disease-free survival (DFS) and OS (42), suggesting its prognostic value in HCC. Besides, the previous studies based on the bioinformatics analysis reported that *MAPT* (43), *BIRC5* (44), *PLXNA1* (45), *CHGA* (46), and *SPP1* (37) were used for the construction of the prognostic model and probably influenced the OS time of the patients with HCC. Since the results have not yet been validated externally, our analyses add further credibility to these findings.

The constructed risk model was then utilized to distinguish the high- and low-risk group among the patients with HCC. Subsequently, the univariate and multivariate regressions were employed to analyze the differences in OS between the two groups and the results demonstrated that the survival time of the two risk groups differed significantly indicating the effective prognostic value of the signature.

It is noteworthy to mention that the DEGs selected in accordance with the different risk groups are involved in the tumor-related pathways such as ECM–receptor interaction and IL-17 signaling pathway. Studies have found that not only ECM has been proved to be essentially responsible for the promotion of the invasion, metastasis, and EMT process of the cancer cells (47–49), but changes in its composition promote the cancer formation and progression as well as mediate drug resistance by blocking effective drug delivery (50–52). For example, the elevated levels of the matrix metalloproteinases (MMPs) are usually associated with an undesirable prognosis and a higher risk of recurrence in the breast cancer (53). Tumor-associated macrophages (TAMs) have been found to directly result in the degradation of ECM and promote the invasion of the tumor cells by secreting the proteolytic enzymes (MMP-2 and MMP-9) and stromal-associated proteins (51, 54). Moreover, the infiltrated macrophages secrete the enzymes and cytokines to promote ECM stiffness (55), which, in turn, lead to the tumor proliferation, migration, invasion, and drug resistance (51). With respect to the role of the inflammatory cytokines IL-17 signaling pathway, the mounting data showed that IL-17 positively correlated with the tumor proliferation, progression, and metastasis in various malignancies such as prostate cancer (56), colorectal cancer (57), lung cancer (58), and HCC (59).

To investigate the relationship between the risk scores and immune cells infiltration, we analyzed the tumor immune infiltration signatures in the tissues of HCC, concluding that infiltrating the NK cells, Treg cells, and macrophages may be involved in the development of cancer. Recent attentions have been paid to the role of the tumor-infiltrating immune in cancer. Current chemotherapy and radiotherapy regimens can promote the antigen exposure on the tumor surface, thereby stimulating the accumulation of the Treg cells (60). However, the abundance of the Treg cells in the tumor tissues is generally associated with the poor clinical prognosis. In contrast, depletion of the Treg cells can effectively improve to activate the anticancer immunity and NK cell proliferation. For example, the anti-CTLA4 (61) and anti-CCR4 (62) antibodies have been shown to reduce the Treg cells infiltration and enhance antitumor immune responses. Similarly, chemotherapy also increases the infiltration of the TAMs in the tumor tissues and the activated TAMs also make promotion on the tumor progression by secreting proinflammatory cytokine such as interleukin-6 (IL-6) (63). Depletion of the TAMs and inhibition of differentiation to M2 phenotype significantly enhance the antitumor effects of chemotherapy by activating the antitumor T-cell responses (64, 65). Different from the Treg cells and macrophages, NK cell infiltration is associated with longer survival time and is expected to enhance the antitumor responses (66). Interestingly, many novel targeted drugs are designed to exert their antitumor activity by the NK cell-mediated antibody-dependent cell-mediated cytotoxicity (ADCC) (67). The interaction between the immune cells and the corresponding enrichment pathway is expected to be a potential anticancer therapeutic target.

Previous studies only evaluated the infiltration status of the immune cells in the TME. However, they did not explore the specific functions in which these cells are involved. IFN-I and IFN-II responses are known to be involved in antitumor immune response by activating the NK cells, suppressing the activity of the Treg cells, and the differentiation to M2 of TAMs (68). In this study, the immune cells (such as the NK cells, Treg cells, and macrophages) might be involved in the cancer immunity through modulating IFN-I. IFN-I is generally thought to promote the cytotoxic T lymphocytes (CTLs) antitumor responses and suppress the proliferation of the cancer cells (69, 70). On one hand, IFN-I can increase the cytotoxicity of the NK cells and the CD8+ T cells against the tumor cells (71). On the other hand, IFN-I is capable of prolonging the survival time of the CD8+ T cells and protect them from attacking the natural cytotoxicity receptor 1 (NCR1)-mediated NK cells (72, 73). In addition, IFN-I can decrease the immunosuppression of the Treg cells by upregulating phosphodiesterase 4 (PD4) and downregulating cyclic AMP (cAMP) (74). Moreover, IFN-I signaling is able to enhance the inflammation responses by the macrophages through regulating the secretion of the IL-1β and IL-18, promoting antitumor immune response (75). Nevertheless, there have been evidences that IFN-I also exerts the immunosuppressive effects. IFN-I can upregulate the abundance of the Treg cells and promote indoleamine 2,3-dioxygenase (IDO) expression (an immunosuppressive enzyme produced by the macrophages) and PD-L1 (an IFN-I-responsive gene that suppresses CTL activity) as well as the level of the checkpoint antagonists that suppress the antitumor immune responses (76–80). IFN-I signals may have opposite endings under the different conditions; therefore, the mechanism of IFN-I in antitumor immune response needs further external verification.

Lastly, based on the constructed model, IC50 values were calculated to assess the chemosensitivity of drugs authorized by the AJCC. As a result, cisplatin, doxorubicin, etoposide, gemcitabine, and mitomycin C seem to be more suitable for the treatment of the patients with HCC with the high-risk scores. However, sorafenib, approved as the first-line treatment option for HCC nowadays, did not show any significant superiority in the high- or low-risk groups. These results could provide a direction for the clinical trials that evaluate the applicability of these therapies.

In comparison with the recently published studies (81), this study selected the six EMT-related genes that are different from Bian et al. study and tried a novel and similar algorithm (Lasso regression) to construct the risk assessment model. We divided the patients with HCC into the two risk populations and further revealed the correlations between the immune infiltration status and risk scores as well as assessed the chemosensitivity of the approved drugs for HCC. Nevertheless, there are a few shortcomings in this study. First and foremost, our model was established and verified based on the public datasets (the TCGA and the ICGC) but lacked experimental data to validate. Furthermore, the raw data for all of the analyses were relatively insufficient; hence, it is necessary to increase the sample size in the future studies. Moreover, the potential mechanisms of IFN-I and IFN-II in the antitumor immunity need to be further explored. Last but not least, this study is presently based on the signal-dimensional analyzing frame. Transcriptome level data mining methods have been improved but the development of multi-omics approach has shown potential in the future. The application of single-cell multi-omics technique helps to provide a more complete map of the gene regulatory networks in the complex tissues. Through the effective multi-omics analysis relations among the public datasets, the optimal models will be able to be constructed in order to improve the predictive performance (82–85).

## Conclusions

In conclusion, this study constructed the six-genes-based signature that has a great predictive value for the prognosis of HCC. Based on this signature, we found that infiltration of the NK cells, Treg cells, and macrophages was significantly associated with the high-risk scores and IFN might be involved in the progression of HCC. In addition, we also provided a reference for the clinical selection of the authorized chemotherapy drugs in the different populations. The underlying mechanisms of the immune infiltration deserve further exploration.

## Data Availability Statement

The datasets presented in this study can be found in online repositories. The names of the repository/repositories and accession number(s) can be found in the article/Supplementary Material.

## Author Contributions

GW, YY, and YZhu contributed to the conception and design. YZho and QG contributed to the administrative support. YY, YL, ZZ, and LA contributed to the provision of the study materials or patients. GW, ML, YZhe, and YW contributed to the collection and assembly of the data. GW, YY, and YZhu contributed to the data analysis and interpretation. All the authors contributed to the writing and final approval of the manuscript.

## Funding

This study was supported by the National Natural Science Foundation of China (71964021, 81960430), the Natural Science Foundation of Gansu Province (21JR1RA117), and the Hospital Fund Project of the First Hospital of Lanzhou University (ldyyyn2018-38, ldyyyn2020-50).

## Conflict of Interest

The authors declare that the research was conducted in the absence of any commercial or financial relationships that could be construed as a potential conflict of interest.

## Publisher's Note

All claims expressed in this article are solely those of the authors and do not necessarily represent those of their affiliated organizations, or those of the publisher, the editors and the reviewers. Any product that may be evaluated in this article, or claim that may be made by its manufacturer, is not guaranteed or endorsed by the publisher.
