# Supplementary material for: Comprehensive Analysis to Identify the Epithelial–Mesenchymal Transition-Related Immune Signatures as a Prognostic and Therapeutic Biomarkers in Hepatocellular Carcinoma
Source: Front Surg. 2021 Oct 15;8:742443. doi: 10.3389/fsurg.2021.742443 (PMC8554059; doi:10.3389/fsurg.2021.742443)
Supplement: Supplementary file 1 [file Data_Sheet_1.docx]

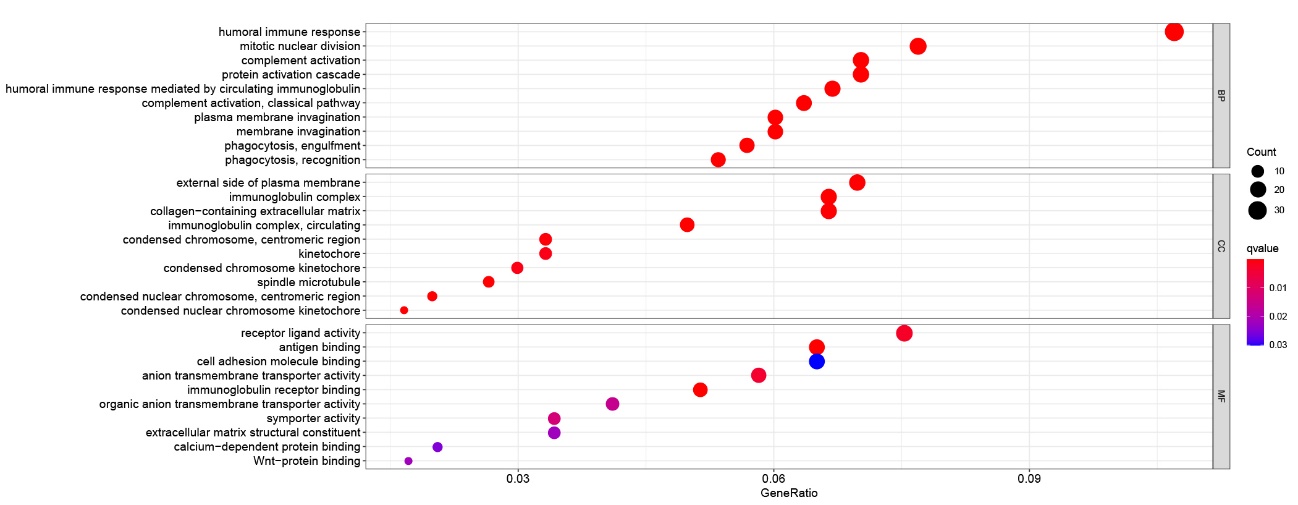


**Figure S1 Bubble plot of GO analysis based on TCGA dataset.**


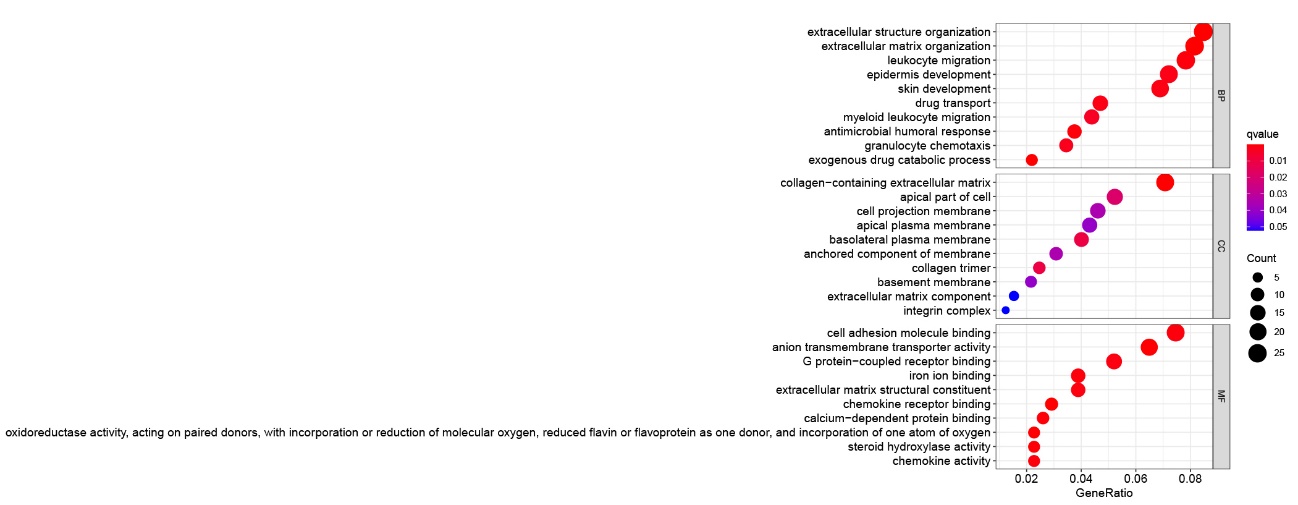


**Figure S2 Bubble plot of GO analysis based on ICGC dataset.**


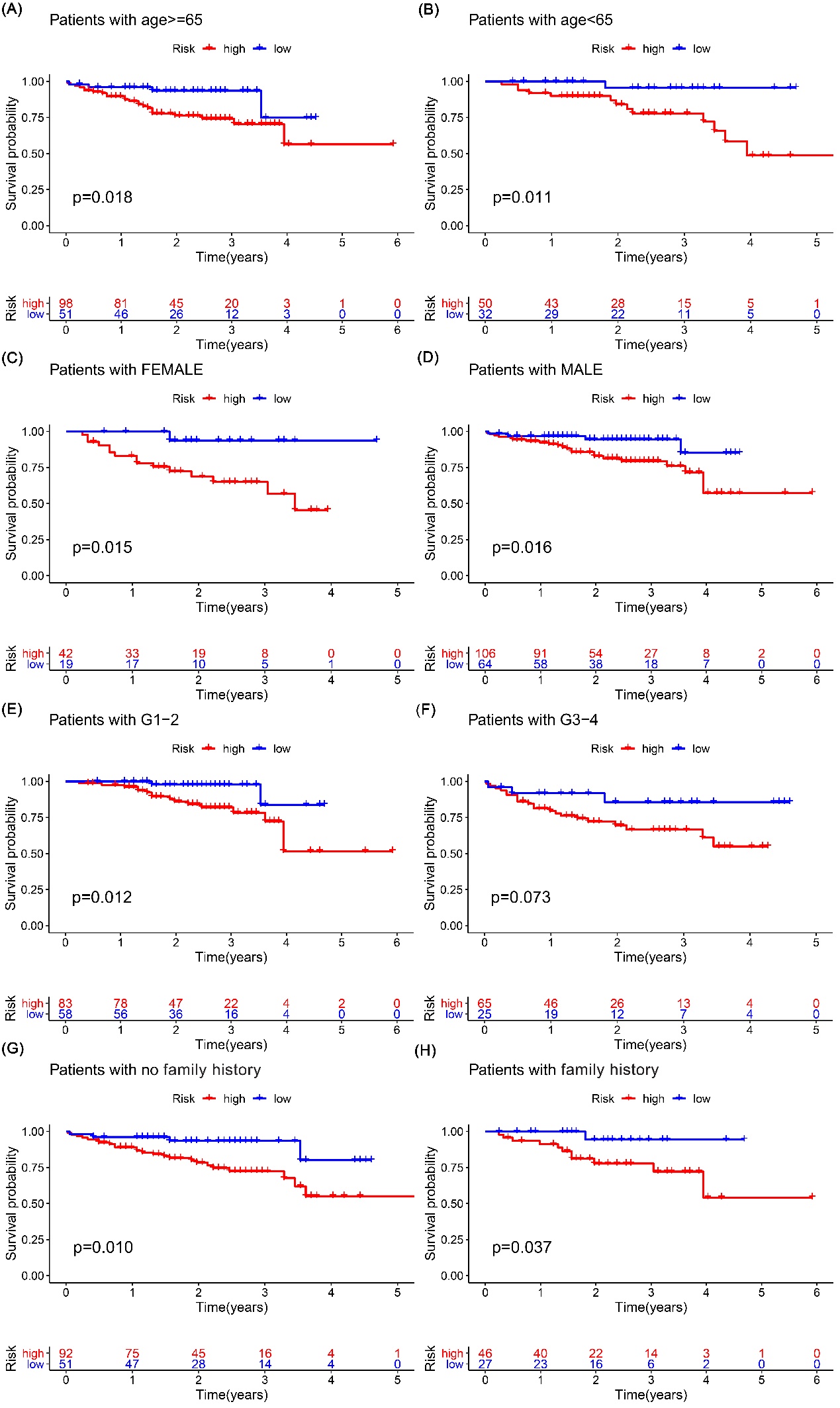


**Figure S3 Stratified analysis validated by ICGC dataset.**

Survival curves show higher survival probabilities of low risk group compared with high risk group stratified by (A) age≥65, (B)＜65, (C) female, (D) male, (E) G1-2, (G-H) Stage I-IV, while no significantly statistical difference was found after stratification by (F) G3-4.


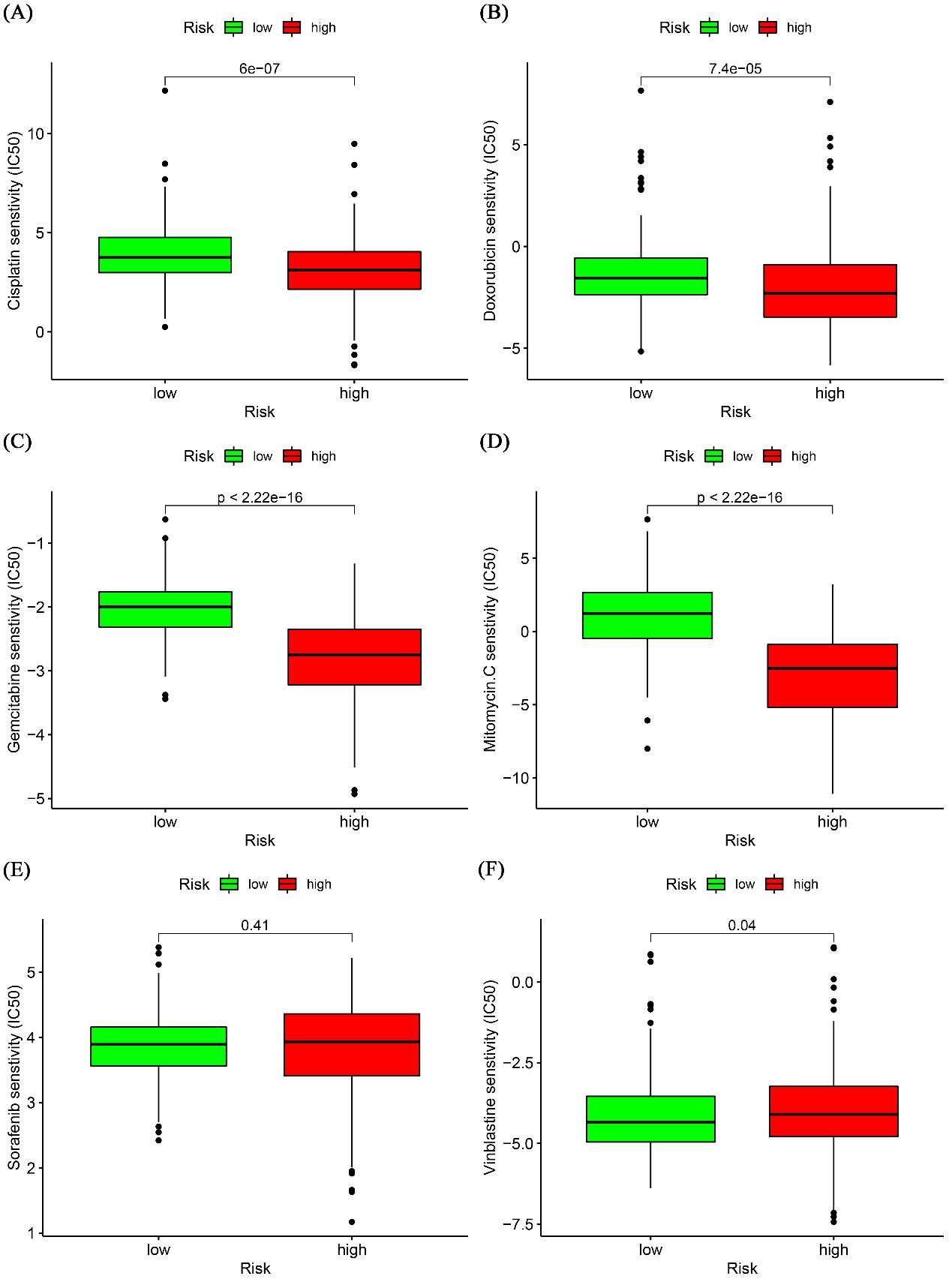


**Figure S4 Predication of chemosensitivity based on risk model.**

The model showed risk score was positively correlated with chemosensitivity of (A) Cisplatin, (B) Doxorubicin, (C) Gemcitabine, (D) Mitomycin C, but negatively associated with (E) Vinblastine and (F) Sorafenib.
